# Public Attitude towards Implementing DNAR and a Stance on Pediatric DNAR in Poland—A Prospective Survey Study

**DOI:** 10.3390/jcm13061755

**Published:** 2024-03-18

**Authors:** Dariusz Timler, Joanna Kempa, Remigiusz Kozłowski, Robert Stolarek, Wojciech Timler

**Affiliations:** 1 Emergency Medicine and Disaster Medicine Department, Medical University of Lodz, 90-419 Lodz, Poland; dariusz.timler@umed.lodz.pl (D.T.); robert.stolarek@umed.lodz.pl (R.S.); 2 Individual Course of Study in Plastic, Reconstructive and Aesthetic Surgery Clinic, Institute of Surgery, Medical University of Lodz, 90-419 Lodz, Poland; joanna.kempa@stud.umed.lodz.pl; 3 Department of Management and Logistics in Healthcare, Medical University of Lodz, 90-419 Lodz, Poland; remigiusz.kozlowski@umed.lodz.pl; 4 Department of Family Medicine, Medical University of Lodz, 90-419 Lodz, Poland

**Keywords:** do not attempt resuscitation, do not resuscitate, cardio-pulmonary resuscitation, ethics, euthanasia, palliative care, taboo

## Abstract

**Background:** Do not attempt resuscitation (DNAR) is a document signed by a patient, which states that they do not want to be resuscitated. In Poland, DNAR is not regulated by law. We aimed to assess people’s perceptions on DNAR and pediatric DNAR in Poland. **Methods:** An anonymous survey was distributed via the snowball sampling method in different voivodeships in Poland in the years 2014–2018. The survey consisted of questions regarding knowledge and attitudes towards DNAR and pediatric DNAR. **Results:** A total of 1049 responses were collected. Moreover, 82% support introducing DNAR in Poland, but 78% believe that this is not a pressing issue. In a general question, 46% of respondents believe that DNAR should be obtainable only for adults. However, in a specific question, this number drops to 17%, with people agreeing for pediatric DNAR if it contains a boundary—23% agree if both parents agree to the solution and 45% if both parents and the child’s doctor agree to it. **Conclusions:** Even though someone supports DNAR, it does not mean that they support pediatric DNAR. People outside the medical community are more likely to be against DNAR. Giving a boundary in using pediatric DNAR may lead to the ease of its implementation in a legislative manner.

## 1. Introduction

Do not attempt resuscitation (DNAR) is a medical document in which a patient states that, in case of the event of cardiac and/or respiratory arrest, they do not want to be resuscitated [1]. When a patient is terminally ill and in a lot of pain, they might not want to have their life prolonged by needless cardio-pulmonary resuscitation (CPR). Some people, however, consider this practice to be on par with euthanasia—bringing death to another person because of the belief that they would be better off dead [2,3]. This naturally is a controversial statement as DNAR is passive and results in not helping, while euthanasia is mostly an action that brings death in a controlled way to a person [4].

In Poland, the abandonment of resuscitation is rare because DNAR is not regulated by law. Resuscitation withdrawal is supported by ethics codes in which there is a regulation on so-called persistent therapy [5]. When it comes to the issue of pediatric DNAR, it is not only used very rarely, but also almost no person will speak about it in the clinic—it falls under taboo topics in east European culture.

This study, to the best of our knowledge, is the first survey and analysis of current opinions on do-not-resuscitate orders in Poland. It is original even across all Eastern Europe regions. The consequences of a do-not-resuscitate order have been quite thoroughly studied in countries where DNR orders were implemented. This is the first original attempt to survey both medical and non-medical professional opinions on DNR orders in adult and pediatric patients in Poland.

The aim of the study is to highlight the problem of the lack of regulation about DNAR in Poland, as well as in most of Eastern European countries. The issue of DNAR among adults is already controversial, and in children, it is an even more complicated problem, mainly due to the fact that such a decision must be carried out by the parents. Because the topic keeps evoking ethical and moral problems, we decided to collect the opinions of Poles, which could result, in the future, in the introduction of pediatric DNAR legislation in Poland.

## 2. Materials and Methods

An anonymous online survey created with Google Forms was distributed using the “snowball sampling” method via e-mail between the years 2014 and 2018 among people living in various regions of Poland and of miscellaneous professions and age. The minimal sample size of the study equaled 385 respondents, and it was calculated using a sample size calculator online with the assumptions being a 95% confidence level, 5% margin of error, 50% population proportion and 37,750,000 population size (roughly equal to the Polish population at the time of data collection). A chi-squared statistical power calculator online was used in order to determine the test power with the assumptions of α = 0.05, medium effect size (0.3), 8 categories (as many as the answers to the question “Are You in favour of implementing DNAR for children?”) and a 385 sample size. The test power with these assumptions equaled 0.9972.

An email consisting of the survey was sent to 10 people living in each Polish voivodeship capital city (160 people in total). These people were asked to forward the message to 5 different people living in their voivodeship with a request to forward it further. The questionnaire consisted of multiple open and one-choice questions, regarding demographic data, living environment, knowledge and attitudes towards DNAR and pediatric DNAR.

The process of creating and evaluating the questionnaire was as follows:

Stage 1. Literature review to identify a range of problems used in research on similar topics, including literature reviews, original articles, guidelines and international recommendations.
Stage 2. Preparation of a list of questions appropriate to verify the chosen aims of the study.
Stage 3. Evaluation of the content by medical professionals (n = 10) and non-medical participants (n = 10)
Stage 4. Implementing improvements suggested by people who took part in study in Stage 3.
Stage 5. Completing the evaluation of the questionnaire.

The exclusion criteria were not living in Poland, not possessing a Polish citizenship and an age below 18. The main condition for participation in the study was the completion of the questionnaire.

The analysis was carried out using the Statistica 13.3 program. Elements of descriptive statistics were used to determine the percentage (%) and standard deviation (SD). The Pearson chi-squared test was used in order to compare the answers of different groups against one another—gender, size of the city they live in and profession.

## 3. Results

A total of 1049 questionnaires were collected with the respondents’ mean age being 34.42 ± 13.01 years, where 414 (39.47%) were male and 635 (60.53%) were female. Moreover, 250 (23.83%) declared themselves to be a pupil/student, 756 (72.07%) were at their working age and 43 (4.1%) were retired. The respondents came from multiple living areas from different parts of Poland, although most of them, 524 (49.95%), came from a big city (over 400 thousand people). Furthermore, 363 (34.6%) were not working in a medical field, 190 (18.11%) were medical doctors, 213 (20.31%) were medical students, 114 (10.86%) were nurses, 123 (11.73%) were paramedics and 44 (4.2%) performed other medical professions. All of respondents’ characteristics can be visible in Table 1.

Overwhelmingly, 82% of the respondents consider themselves to be supporters of introducing DNAR in Poland (n = 422 strongly for introducing DNAR, and n = 439 rather strongly for introducing DNAR). At the same time, only 10% (n = 105) think that it is actually necessary, with an overwhelming 78% (n = 816) believing that it is not needed in Poland right now.

**Figure 1:**
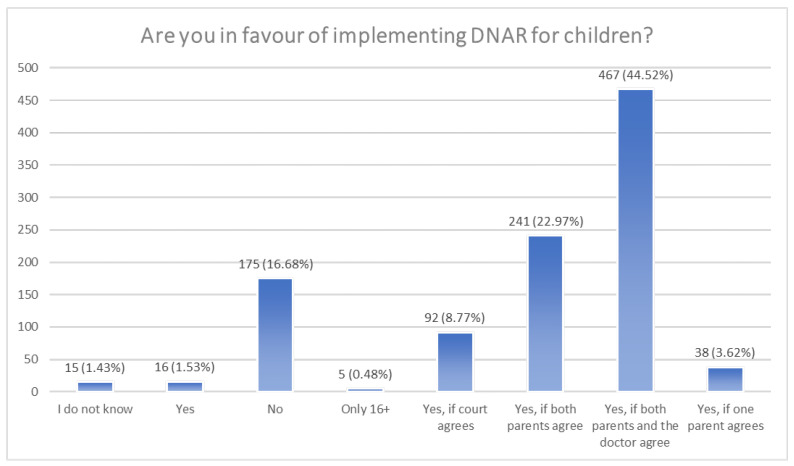
Are you in favour of implementing DNAR for children?

When it comes to the question of accepting DNAR only for adults, the answers are mixed: 33% (n = 349) are for implementing it for children as well, whereas 46% (n = 485) claim that it should be available only for grown-ups, with the rest staying neutral on the topic (n = 215). However, the response changes when we ask about DNAR for children in detail (Figure 1). Suddenly, only 17% (n = 175) are flat out against introducing it, with most answers directed towards agreeing with some sort of boundary. The most popular options are as follows: 44.5% (n = 467) accept DNAR for children in the case where both parents and the child’s doctor agree; 23% (n = 241) in the case where both parents agree; and 9% (n = 92) in the case where the court gives permission.

While analyzing the answers of only the supporters of DNAR (n = 861), 29.2% (n = 251) declared that it should be available not only for adults but also for children, while 50.4% (n = 434) claimed that it should be acceptable only among adults. When asking DNAR supporters in detail, only 9.3% (n = 80) were against implementing it among children, and 50.5% (n = 435) indicated that DNAR should be accepted if both the parents and the child’s doctor agree. The other popular options, with 24.5% (n = 211) approval, were accepting DNAR if both parents agree and 8.3% (n = 71) accepting it if the court agrees.

Out of the 115 respondents who are against introducing DNAR in Poland, 60.9% (n = 70) declared that it should be acceptable also for children, while 25.2% (n = 29) thought that it should only be acceptable among adults. When asked in detail, 59.1% (n = 68) rejected the idea of implementing DNAR for children, followed by 15.7% (n = 18) of people who claimed that both the parents and child’s doctor must agree to proceed with DNAR.

Comparing the answers of the two groups mentioned above—people in favour of DNAR (n = 861) and people against DNAR (n = 115)—to the question “Would you want to have DNAR implemented for children?”, using Pearson’s chi-squared test (*p* = 0.0000), there were statistically significant differences between the answers, where DNAR supporters responded no to implementing DNAR for children less than people who were against DNAR. The same situation applies for the answer “the court has to agree”. DNAR supporters voted more often for that question with the response of “yes, but both parents have to agree” and “yes, but both parents and the childs’ doctor have to agree” than people against DNAR.

The group of people who think that DNAR is necessary in Poland comprised 105 people, in which 57.1% (n = 60) said they would not want DNAR implemented for children. Moreover, 16.2% (n = 17) of people from that group thought that DNAR for children is acceptable if both parents agree, 15.2% (n = 16) if both parents and the child’s doctor agree and 9.5% (n = 10) if the court agrees.

The group of people who think that DNAR is not necessary in Poland comprised 816 people, from whom 8.5% (n = 69) would not want DNAR implemented among children. Moreover, 50% (n = 408) of people from this group think that DNAR should be implemented for children only if both parents and the child’s doctor agree, 25.3% (n = 206) only if both parents agree and 8.3% (n = 68) only if the court agrees.

Comparing the answers of the two groups mentioned above to the question “Would you want to have DNAR implemented for children?”, using Pearson’s chi-squared test (*p* = 0.0000), people who think that DNAR is necessary in Poland (n = 105) voted more often against DNAR implementation for children than people who deem it unnecessary (n = 816). People who think that DNAR is unnecessary in Poland were more likely to vote for the options “yes, but both parents have to agree”, “yes, but both parents and the childs’ doctor have to agree”, and “yes, only one parent needs to agree” than people who think that DNAR is necessary in Poland.

While analyzing the differences in answers between sex, it turned out that women more often think that implementing DNAR in Poland is unnecessary. No statistically significant difference was found when analyzing the size of the city where respondents were living.

The occupation of the respondents was an important factor that influenced the answers. Comparing the answers of medical professionals (n = 684) and people who do not work in a medical field (n = 363) to the question “Would you want to have DNAR implemented for children?”, using Pearson’s chi-squared test (*p* = 0.0005), it turned out that medics were against DNAR implementation for children less often than people who are not working in a medical field (Table 2). Medics were more likely to choose “yes, but the court has to agree” and “yes, but both parents and the childs’ doctor have to agree” than people whose occupation was not related to medicine.

Medics were more likely to answer that implementing DNAR is not necessary in comparison with non-medics (*p* = 0.0089). Nurses (n = 114) declared more often that DNAR is not necessary than medical students (n = 213) and people who do not work in a medical field (n = 363) (*p* = 0.0008). Medical doctors (n = 190) answered that DNAR should be implemented only for adults more likely than non-medics and students (*p* = 0.0001).

## 4. Discussion

Do not attempt resuscitation (DNAR) is a declaration from patients or patients’ legal guardians (if the patient is under 18 years old or needs a legal guardian declared by law) which gives permission for medical workers not to perform CPR if the patient needs it. The main goal of it is to avoid suffering. Usually, patients decide to sign DNAR forms before high-risk operations such as neurosurgical operations which may lead to a coma or a vegetative state. Other reasons for deciding to sign it is when a person is in the terminal stage of a chronic illness which is uncurable, so the performance of CPR, if needed, will only prolong the suffering of the patient for some time [6,7]. The use of the DNAR protocol is more prevalent among emergency patients and is associated with age and non-trauma presentation [8]. Polish law lacks regulations on persistent therapy. There is only an ethical rule that exempts the doctor from the obligation to conduct it. In contrast, there are no regulations that mandate respect for the patient’s will, expressed in the event of unconsciousness, and ensure that it is enforced [5]. This is worrying and in need of changing as even though the patient claims not to want to be resuscitated, the family might still challenge the lack of CPR in court. Because of this, many doctors refrain to exercise the patients’ will, as they fear legal repercussions later on.

While talking about DNAR, most people think about a very old and ill person, but it does not refer only to them. The topic is unique while thinking about children. As mentioned earlier, the decision regarding DNAR in pediatrics is not made by a patient, but by a legal guardian. Children are generally more vulnerable and dependent on adults for their care and well-being. They lack the physical and emotional maturity to navigate the world on their own, making them more in need of protection and support. This vulnerability can evoke an instinct to care for and protect them, and that is why the level of compassion towards children is higher than those toward adults [9]. Therefore, a person that supports DNAR implementation for adults may not feel the same towards pediatric DNAR. In our study, 33% (n = 349) are for implementing DNAR for children (not only adults), whereas 46% (n = 485) claim that it should be available only for grown-ups, and the rest stayed neutral. Some of these people were against the concept in general. However, it is still surprising that the majority would oppose it, just because of the change in the target group. Hoehn et al. (2009) conducted a survey about implementing DNAR in pediatric patients, and 96% of respondents were highly supportive of respecting the parents’ request for DNAR [10]. However, pediatricians who took part in the previously mentioned study were more knowledgeable about diseases and their outcome in young patients, which had a significant impact on the survey results. In our research, almost 35% were people who had nothing to do with medical work, which might have a big influence on their opinion because they did not have the opportunity to observe children’s illnesses as often as people working in hospitals.

A very eye-catching change in responses happened when the questions changed from broad to more specific—when given some sort of boundary, suddenly, only 17% of respondents answered negatively compared to 46% when asked in general. The most popular choice of the boundary was consent from both the parents and the child’s doctor. This and other restrictions may prevent DNAR being given too loosely. However, they may also result in a blockade of DNAR when it would be preferable. A study from Japan points out several key advantages that derive from having a DNAR order, such as the ease of response and unified intention from the team. This order might also relieve some pressure from parents of the child put into a DNAR procedure—most commonly, reports have stated that DNAR was not fully understood by all family members [11]. This is particularly worrying and shows the need for change. The majority of patients are documented to be willing to talk about it, especially prior to them being critically ill, as well as involving their family members in the decision-making process [12]. On the other hand, a study from 2020 points out that cancer patients, who received DNAR explanations during their therapy, had a worse prognosis than those who had no records of talking about it [13].

The vast majority of participants in the study support DNAR in Poland, and almost the same amount of the persons surveyed declare that there is no need to implement DNAR right away in this country, thus demonstrating hesitation or the lack of firm opinion. Therefore, it would be interesting to compare spontaneous responses to the same survey questions after providing some educational materials of DNAR medical cases or televised expert debates. This could possibly affect lay people’s responses and make their response to survey questions more like medical professionals. The issue of accepting DNAR only for adults provided equally mixed responses, indicating probably the quite random choice of those surveyed. Also, women in our study more often declared that implementing DNAR in Poland is unnecessary. It should be emphasized that a lot of those initially opposing DNAR in children change their mind if given some sort of boundary.

What is perhaps most surprising in the results is that medics are more opposed to DNAR in comparison to non-medics. One might think that medical procedures and legislations such as DNAR forms are popular among people working in medical fields. However, it turns out that many medics are very traditional and do not like big changes, which might explain the results of this question. However, it is widely known that accepting new procedures can improve patient care and even the hospital/clinic finances [14].

Another reason why medics declared DNAR to be unnecessary more than non-medics might be that they know how the hospital functions and that this procedure is performed in hospitals, even though there is a lack of legislation. However, even if it actually happens, it would only cover the needs of elderly patients as not performing CPR in young adults and children would definitely end up in court as there are no specific guidelines to refer to [15].

An interesting finding is that medical doctors are more likely to vote against allowing DNAR for children in comparison to students and non-medics. A big survey study performed on pediatricians showed a contrarian tendency, with over 80% respecting DNAR and over 75% of them recommending it [10]. Neonatologists, however, even though over 60% of them would respect DNAR, only half of them would put this order on a child when survival is felt to be unlikely [16].

This study describes spontaneous responses to particular medical and ethical problems in do-not-resuscitate cases. The general knowledge of DNAR medical and ethical pitfalls is determined by available information in the media, incidental contact with specific cases, general education, religious considerations and personal life experience. We may speculate that persons supporting DNAR in Poland could reconsider their opinion if they had larger knowledge on more detailed controversies on implementing DNAR orders. An example of this is the implementation DNAR orders in emergency situations, where verifying DNAR documentation in time is limited. The real controversies of DNAR in a medical emergency could also increase the number of persons against implementing DNAR for children. In a recent study, fewer Eastern European and Asian respondents agreed with withdrawing life-sustaining treatments without consent of patients or surrogates. Religion, years in practice or institution did not affect their agreement, but religiosity, physician specialty and responsibility for end-of-life decisions did [17]. Only one recently published study in an intensive care unit described a relation between a DNAR order in a patient’s documentation and the frequency of both resuscitation procedures and withholding catecholamine treatment in the hours preceding a patient’s death [18]. The complexities of the decision not to perform CPR in pediatric hospital settings were recently described as discrepancies identified between practice and medical records in DNR pediatric patients [19].

In the light of the presented findings, it is necessary to outline the factors that determine the particular response of the participants of the study. Apparently, the medical profession gives vast background to ethical and medical particularities of not undertaking resuscitation. In the case of non-medical professionals, background and general knowledge may determine supporting DNAR. The compassionate appreciation of the difficulty of DNAR orders among medical and non-medical persons is a cornerstone finding of this study. Further studies are warranted to delineate the educational, ethical and procedural details of implementing DNAR in this region of Europe.

## 5. Limitations

The question “Do you think that implementing DNAR is only acceptable among adults?” might have been understood incorrectly by some respondents as implicated by the answers of people who are against introducing DNAR in Poland who voted that DNAR should not only be acceptable among adults. The study was conducted over a 4-year period, which may influence the outcomes as peoples’ opinions develop and change over time. The survey distribution via email might have excluded people living without internet or computers as well as people not knowing how to operate the internet/computer well enough.

## 6. Conclusions

As a result of the considerations and analyses, the following conclusions were formulated:

1. Some people that are initially against allowing DNAR for children might change their mind if given some sort of boundary.
2. Even though someone supports DNAR, it does not mean that they support DNAR for children.
3. Many Poles think that DNAR should be implemented for children if there are regulations and boundaries about using it.
4. People who are not working as medical professionals were much more against DNAR than medics.
5. More research is still needed in this area of study.

## Figures and Tables

**Table 1:** Respondents’ characteristics.

Characteristics of the Respondents n = 1049			
Variable	Subgroup	n	%
Gender	Male	414	39.47
	Female	635	60.53
Age	Mean	34.42	
	Standard Deviation	13.01	
Working Age or Not?	Working Age (Pupil/Student)	250	23.83
	Working Age	756	72.07
	Post Working Age	43	4.10
Place of Living	Village	147	14.01
	City under 40 k	108	10.30
	City 40–99 k	128	12.20
	City 100–199 k	55	5.24
	City 200–400 k	87	8.30
	City over 400 k	524	49.95
Profession	Nonmedical	363	34.60
	Medical Doctor	190	18.11
	Medical Student	213	20.31
	Nurse	114	10.86
	Other medical	44	4.20
	Paramedic	123	11.73
	Missing	2	0.19

**Table 2:** Profession and DNAR among children.

Profession	I Don’t Know	Yes	No	Only 16+	Court Has to Agree	Both Parents Have to Agree	Both Parents and the Doctor Have to Agree	One Parent Needs to Agree	Totals
Nonmedical	2	6	84	1	26	88	139	17	363
Row %	0.55%	1.65%	23.14%	0.28%	7.16%	24.24%	38.29%	4.68%	
MD	6	3	22	0	22	39	89	9	190
Row %	3.16%	1.58%	11.58%	0.00%	11.58%	20.53%	46.84%	4.74%	
Medical Student	4	2	44	2	19	36	101	5	213
Row %	1.88%	0.94%	20.66%	0.94%	8.92%	16.90%	47.42%	2.35%	
Nurse	1	0	16	0	8	26	59	4	114
Row %	0.88%	0.00%	14.04%	0.00%	7.02%	22.81%	51.75%	3.51%	
Other Medical	2	1	2	1	2	12	22	2	44
Row %	4.55%	2.27%	4.55%	2.27%	4.55%	27.27%	50.00%	4.55%	
Paramedic	0	4	7	1	15	38	57	1	123
Row %	0.00%	3.25%	5.69%	0.81%	12.20%	30.89%	46.34%	0.81%	
Totals	15	16	175	5	92	239	467	38	1047 (2 missing)

## Data Availability

The raw data supporting the conclusions of this article will be made available by the authors on request.
